# Orofacial Clefts and Risk Factors in Tehran, Iran: A Case Control Study

**Published:** 2012-01-01

**Authors:** N Taghavi, M Mollaian, P Alizadeh, M Moshref, Sh Modabernia, A R Akbarzadeh

**Affiliations:** 1 Department of Oral and Maxillofacial Pathology, Shahid Beheshti University of Medical Sciences, Tehran, Iran; 2 Department of Pediatrics, Tehran University of Medical Sciences, Tehran, Iran; 3 Dental Student, Shahid Beheshti University of Medical Sciences, Tehran, Iran; 4 Department of Basic Sciences, School of Rehabilitation, Shahid Beheshti University of Medical Sciences, Tehran, Iran

**Keywords:** Orofacial cleft, Risk factor, Iran

## Abstract

**Background:**

Non-syndromic cleft lip with or without cleft palate (CL/P) or cleft palate only (CPO) are orofacial clefts with multifactorial etiology. These include environmental factors and heterogeneous genetic background. Therefore, studies on different and homogenous populations can be useful in detecting related factors. The aim of the present study was to evaluate the risk factors in patients with non-syndromic cleft in Tehran, Iran.

**Methods:**

Data from 300 patients and 300 controls were collected between 2005 and 2010. Binary logistic regression analyses were used to calculate relative risk by odds ratio (OR) and %95 confidence interval.

**Results:**

Low maternal age (OR=1.06, 95% CI, 1.011-1.113), low socioeconomic status (OR=0.23, 95% CI, 0.007-0.074), maternal systemic disease (OR=0.364; 95% CI, 0.152-0.873) and passive smoking (OR=0.613, 95% CI, 0.430-0.874) increased the risk for CL/P and CPO. There was a significant difference in iron and folic acid use during pregnancy when the case and control groups were compared.

**Conclusion:**

In assessing for orofacial cleft risk, we should consider lack of folic acid supplementation use, maternal age and systemic diseases and passive smoking as risk factors.

## Introduction

Orofacial clefts are among the most common types of major birth defects, occurring in an estimated 1.5 to 2 per 1000 births.[1] In the United States, approximately 7500 infants are born with cleft malformation each year, subdivided anatomically into cleft lip with or without cleft palate (CL/P) and cleft palate only (CPO).[2] Researchers have proposed several theories to explain the origin of oral cleft. These include environmental factors and heterogeneous genetic background (single genes, gene-gene interactions and gene-environment interactions). Therefore, studies on different and homogenous population can be useful in detecting potentially related environmental and genetic factors.[3][4][5][6]

The aim of the present study was to evaluate whether many factors such as pregnancy exposure (smoking, medication, vitamin supplementation, x-ray), familial history and demographic characteristics were associated with specific types of cleft in a group of patients affected by non-syndromic clefts in Tehran, Iran.

## Materials and Methods

A hospital-based case-control study was performed in Tehran, Capital of Iran. Cases were patients with 0-48 months of age presenting CL/P or CPO not associated with any other birth defects or syndrome (non-syndromic oral cleft). All cases were chosen from Bahrami Hospital, "a reference Pediatric Surgery Unit for orofacial clefts treatment" during 2005-2010.

Controls were a random sample of patients admitted to the same hospital without any birth defects or systemic disease. Mothers of both case patients and controls were interviewed in the hospital by the same investigators. The standardized questionnaire was used to investigate information on the demographic characteristics, socioeconomic status, mother's medical history and pregnancy exposure including tobacco use (active and passive), radiation, folic acid and iron use during pregnancy.

The interview included detailed questions on tobacco use. All mothers were asked whether they ever smoked and if so, whether they have smoked cigarettes any time from 3 months before pregnancy until delivery. Mothers who reported any smoking in this period were asked specifically whether they smoked during these periods: 3 months before pregnancy, and the first, second and third trimester of pregnancy. Mothers were also asked to report the number of cigarettes smoked daily during each of these periods.

Moreover, mothers were asked about their exposure to environmental tobacco smoke (ETS) at home or work during pregnancy. Our assessment of ETS was limited to non-smoking mothers, defined as mothers who reported no active smoking. Analysis was limited to mothers who completely answered all questions.

The research protocol was reviewed and approved by Ethical Committee of Shahid Beheshti University of Medical Sciences. The association between oral clefts (CL/P and CPO) and variables including sex, maternal age, maternal education, socioeconomic status, pregnancy exposure, maternal systemic diseases and consanguinity was assessed by binary logestic regression model.

We estimated odds ratios to evaluate the strength of association. To assess goodness of model, Hosmer and Lemeshow test was used. %95 confidence interval (CI) for odds ratios associated with explanatory variables was considered. Regarding the special distribution of the two variables of folic acid use and familial history, association between these variables and risk for oral cleft was evaluated using Chi-Square test. In this investigation, type I error was considered as α=5%.

## Results

The study included 300 cases of non-syndromic cleft and 300 healthy controls with no birth defects. The distribution of demographic and socioeconomic factors, pregnancy exposure, and familial history of cleft and maternal systemic disease of both groups were presented in Table 1.

**Table 1:** Prevalence of clinical charactristcs (and percent) of predisposing factors of orofacial

**Characteristics**	**Case**	**Control**
**Sex**		
Boy	156(%52)	169(%56/3)
Girl	144(%48)	131(%43/7)
**Maternal age at delivery**		
<20	28(%9/3)	20(%6/7)
20-23	58(%19/3)	38(%12/7)
24-27	127(%42/3)	131(%43/7)
28-31	79(%26/3)	95(%31/7)
32-34	5(%1/7)	7(%2/3)
>34	3(%1)	9(%3)
**Maternal education**		
Illiterate	7(%2/3)	3(%1)
Primary school	71(%23/7)	54(%18)
High school	107(%35/7)	83(%27/7)
Diploma	110(%36/7)	146(%48/7)
University	5(%1/7)	14(%4/7)
**Parental consanguinity**		
Yes	34(%11/3)	25(%8/3)
No	266(%88/7)	275(%91/7)
**X-Ray exposure**		
Yes	7(%2/3)	8(%2/7)
No	293(%97/7)	292(%97/3)
**Monthly salary (Dollar)**		
>250	74(%27/7)	22(%7/3)
250-400	203(%67/7)	181(%60/3)
401-550	18(%6)	69(%23)
<550	5(%1/7)	28(%9/3)
**Iron use**		
Daily use	241(%80/3)	202(%67/3)
No use	59(%19/7)	68(%22/7)
Unknown	-	30(%10)
**Folic acid use**		
Daily use	281(%93/7)	297(%99)
No use	19(%6/3)	3(%1)
**Supplemental vitamin use**		
Daily use	245(%81/7)	267(%89)
No use	55(%18/3)	33(%11)
**Systemic disease**		
Yes	18(%6)	8(%2/7)
No	282(%94)	292(%97/3)
**Mother employment status**		29(%9/7)
Yes	42(%14)	271(%90/3)
No	258(%86)	
**Father employment status**		
Yes	284(%94/7)	295(%98/3)
No	16(%5/3)	5(%1/7)
**Maternal smoking**		
Mother never smoked	293(%97/7)	295(%98/3)
ex-smoker	7(%2/3)	5(%1/7)
**Passive smoking**		
Yes	113(%37/7)	80(%26/7)
No	187(%62/3)	220(%73/3)
**History of familial cleft**^a^		
Yes	10(%3)	0(%0)
No	290(%97)	300(%100)

^a^ Clefts, Bahrami Hospital Iran 2005-2010

196 (65.3%) of the cases had CL/P, while 104 (35.7%) cases had CPO. Among patients with CL/P, 68 (22.6%) cases had bilateral cleft, 81 (27%) cases had left unilateral cleft, 44 (14.4%) cases had right unilateral cleft and 3 (1%) cases had central cleft. In CPO patients, 83 (27.3%) cases had complete cleft, while 21 (7.7%) cases had incomplete cleft. 104 males and 92 females were in the CL/P group whereas the CPO group consisted of 50 males and 54 females. There were no significant differences between the case and control group patients with respect to sex, maternal smoking, X-Ray exposure, consanguinity and medication use (p>0.05 for all variables) (Table 2).

**Table 2:** Assessment of variables effect on orofacial cleft

**Variable **	**Adjusted OR**	***P *value^a^**
**Demographic**		
Sex^a^	0.825 (0.593	0.25
Low maternal age^a^	1.06 (1.011	0.016
Maternal education^a^	-	0.017
Illiterate	-	-
Primary school	1.724 (0.419	0.451
High school	1.662 (0.410	0.477
Diploma	2.753 (0.685	0.056
university	5.306 (0.956	0.25
**Socioeconomic status^a^**		0.001
Monthly salary(Dollar)		-
>250	0.23 (0.007	0.001
250-400	0.84 (0.03	0.001
401-550	0.488 (0.158	0.212
<550	-	-
**Pregnancy exposure^a^**		
Iron use	0.435 (0.286	0.001
Supplemental vitamin use	1.235 (0.693	0.474
X-Ray	1.16 (0.415	0.794
Medication	0.467 (0.201	0.078
Maternal smoking	1.516 (0.340	0.817
Passive smoking	0.613 (0.430	0.007
**Maternal Systemic disease^a^**	0.364 (0.152	0.024
**Consanguinity^b^**	0.71 (0.412	0.217

^a^ Logestic regression model, Hosmer and Lemeshow goodness of fit P value=0.360.

^b^ Chi-square test, P value=0.217

All mothers who had reported smoking were ex-smoker in both groups. The proportion who smoked was 2.3% (7 cases) for cases and 1.7% (5 cases) for controls. Both CL/P and CPO were associated with low maternal age (p=0.016), low maternal education (p=0.017), low economic status (p=0.001) and maternal systemic disease (p=0.024).

There was an inverse relation (OR= 0.43; 95% CI, 0.286-0.727) between iron use during pregnancy and risk of oral cleft (p=0.001). There was also an association between familial history of oral cleft and risk of both CL/P and CPO with the same overall risk (p<0.01). Furthermore, data analysis showed a significant difference (p<0.001) in acid folic use between case and control group. Daily use was approximately 7% lower among mothers in the case group which was associated with the increased risk of CL/P and CPO.

Among non-smoking mothers with exposure to any ETS at home or work during pregnancy, the odds ratio was 1.59 with a cleft defect compared to those who did not (OR=0.63; %95 CI, 0.43-0.84). No dose information was available to quantify the level of ETS exposure.

## Discussion

Epidemiological studies on different populations are important for detecting different genetic groups and for demonstrating the role of environmental factors in different geographical areas.[7][8][9]

Any factor that could prevent the facial processes from reaching each other by slowing down migration, multiplication or both of neural crest cells by stopping tissue growth and development for a time or by killing some cells that are already in that location, would cause a persistence of a cleft.[10]

Analysis of data in the present study in Iran showed that folic acid and iron intake during pregnancy would decrease the risk for orofacial cleft which is adjusted for most studies in this field,[11][12][13] but in contrast to Cziele and Hayes reports.[14][15][16] Furthermore, inverse relationship between folic acid intake and risk for orofacial cleft is supported by findings from a large case-control study in which folic acid antagonist (dihydrofolate reductase inhibitors) was shown to increase the risk for orofacial clefts.[17]

There are several lines of evidence suggesting the folate-hemocysteine metabolism to be implicated in the risk of orofacial clefts. However the explanation for this association is unknown but recent information shows endothelial nitric oxide synthase (NOS3) genetic variants expressing NOS3 involvement in folate- homocysteine metabolism which inhibits nitric oxide resulting in hypertension and fetal growth retardation in pregnant rats.[17][18]

It has recently been observed that the different levels of endogenous nitric oxide in different time periods influenced the balance between cell cycle progression and programmed cell death in developing neural plate of thick embryos-cells that contribute to facial development.[17] So we can emphasize the preventive role of folic acid in oral cleft development.

In consistent with Leite et al. study,[3] the present study showed that familial history of oral cleft was significantly associated with both CL/P and CPO which can imply the role of genetic factors. Over the past decades, a considerable interest has developed in the identification of genes that contribute to the etiology of orofacial cleft. The first candidate gene was transforming growth factor-a (TGF-A) which showed an association with non-syndromic cleft lip and palate.[19][20][21]

Evaluation of gene-environment interactions is still in its preliminary stage. Studies of role of smoking in TGF-A and MSx1 genes as covariates have suggested that the risk for orofacial clefting may be influenced by maternal smoking alone as well as combination with the presence of uncommon TGF-A allele.[18] With respect to lack of association between maternal smoking and risk for orofacial cleft in this study in contrast to other studies [4][5][17][20][22][23][24] the current data support the possibility that smoking has a different effect on cleft risk among women which may reflect a role for genetic susceptibility factors in cleft development.

The current study showed ETS exposure increased the risk for orofacial cleft which was similar to other studies.[5][22][23][24][25] The proportion of non-smoking cases reporting ETS exposure was 38% which was higher than control mothers (26%). One limitation was our inability to quantify ETS exposure, but studies using cotinine levels of measured dose of ETS exposure have documented adverse outcome at the highest level of ETS exposure.[25][26]

Low socioeconomic status and low maternal education seemed to be risk factors for having a child with orofacial clefts similar to Kraples et al. study.[25][26] In line with Kraples et al., we speculated that low socioeconomic status as a risk factor should be considered because it can be a marker of parental health and life style. Individuals with low education tend to smoke more and have less healthy diets and nutrients. The life style factors, either alone or combination with occupational activities and genetic background, play a role in the etiology of orofacial cleft.

In line with other studies we have found that low maternal age at delivery[17][18] and maternal systemic disease increased risk for orofacial cleft.[25][26] However we encountered few limitations during recalling and interviewing mothers, while the current study had some notable strength. The large sample size and the location of the study enabled us to extend the results to Iranian population because the center we chose was a referral center for orofacial cleft surgery. Furthermore, we evaluated approximately all of the risk factors that have been considered in other studies separately.[27]

In conclusion, this study demonstrated the role of some environmental factors in this geographic area for orofacial cleft forming. It is known that passive smoking, lack of folic acid and iron use during pregnancy may play role in inducing cleft formation. Moreover, a statistically significant association between low maternal age and education, systemic diseases and familial history of oral cleft and higher risk of CL/P and CPO was observed.
